# Association Between Socioeconomic and Demographic Characteristics and Non-fatal Alcohol-Related Injury in Maringá, Brazil

**DOI:** 10.3389/fpubh.2020.00066

**Published:** 2020-03-25

**Authors:** Deena El-Gabri, Nicole Toomey, Nelly Moraes Gil, Aline Chotte de Oliveira, Paulo Rafael Sanches Calvo, Yolande Pokam Tchuisseu, Sarah Williams, Luciano Andrade, Joao Ricardo Nickenig Vissoci, Catherine Staton

**Affiliations:** ^1^Duke Global Health Institute, Duke University, Durham, NC, United States; ^2^Division of Emergency Medicine, Duke University Medical Center, Durham, NC, United States; ^3^Department of Nursing, State University of Maringá, Maringá, Brazil; ^4^Department of Medicine, Ingá University Center, Maringá, Brazil

**Keywords:** injury, socioeconomic, demographic, alcohol, Brazil

## Abstract

**Background:** Previous research has corroborated a high burden of alcohol-related injury in Brazil and the presence of socioeconomic disparities among the injured. Yet, individual-level data is scarce. To fill this gap, we examined the association between demographic and socioeconomic characteristics with non-fatal alcohol-related injury in Maringá, Brazil.

**Methods:** We used household survey data collected during a 2015 cross-sectional study. We conducted univariate and multivariate analyses to evaluate associations of demographic (age, gender, race) and socioeconomic characteristics (employment, education, income) with non-fatal alcohol-related injury.

**Results:** Of the 995 participants who reported injuries, 62 (6.26%) were alcohol-related. Fifty-three (85%) alcohol-related injuries were reported by males. Multivariate analysis indicated being male (OR = 5.98 95% CI = 3.02, 13.28), 15–29 years of age (OR = 3.62 95% CI = 1.72, 7.71), and identifying as Black (OR = 2.38 95% CI = 1.09, 4.95) were all significantly associated with increased likelihood of reporting an alcohol-related injury, whereas unemployment was significantly associated with decreased likelihood of reporting an alcohol-related injury (OR = 0.41 95% CI = 0.18, 0.88).

**Conclusion:** Our findings suggest that in Maringá, being male, between the ages of 15 and 29, employed, or identifying as Black were characteristics associated with a higher risk for non-fatal alcohol-related injury. Individual level data, such as ours, should be considered in combination with area-level and country-level data when developing evidence-based public-health policies.

## Introduction

In 2016, 2.8 million deaths and 99.2 million disability-adjusted life years (DALYs) were attributed to alcohol use (1). Approximately one-third of all alcohol-attributable DALYs and nearly half of alcohol-attributable deaths are due to injuries that result from alcohol involvement (2, 3). In the lower- and middle-income countries (LMICs) of the Latin American Caribbean (LAC) region, alcohol-related injuries compose a significant portion of the region's global burden of disease and injury, with an estimated one death every 100 s attributed to alcohol misuse (3). Socioeconomic status (SES) is an important risk factor for alcohol-related injury and to the experience of negative alcohol-related consequences (4). While there is substantial previous literature documenting SES risk factors for alcohol-related injuries in high-income countries (HICs), research on the topic in LMICs of the LAC region is scarce (5, 6), and when it exists, it is mostly related to large mega-cities, which deviate from most areas in the LAC countries.

Pooled international studies looking at distribution of socioeconomic disparities and alcohol-related harm SES characteristics and behaviors of alcohol use indicate that patterns of disparities vary greatly across and within countries (6, 7). In HICs, individuals of higher SES are more likely to consume alcohol but tend to do so with greater moderation (and thus less injury risk) (6, 7). In contrast, those of lower SES are more likely to be abstainers, but if they do drink, tend to do so in more harmful ways (6, 7). In Brazil, specifically, previous data indicate high rates of hazardous alcohol use behaviors (i.e., binge drinking, drink driving) across all levels of SES (8, 9). Worldwide, while men are at a higher risk for hazardous alcohol use and associated injury, findings from Brazil note a narrowing of this gap in higher SES strata, as women of higher SES are increasingly reporting alcohol consumption behaviors more similar to men (8–10).

To date, much of the patient-level data on alcohol-related injuries originate from emergency department studies focused on fatally injured patients and from large metropolitan areas, thus are not representative of all regions across Brazil (3, 11). Brazil, as a proxy to many LAC countries, has a heterogeneous socioeconomic distribution by and across regions; more data describing communities outside state capitals or large metropolitan areas is crucial to understand the unique characteristics of alcohol-related injuries. Moreover, these studies only include individuals who seek medical treatment and may not be representative of individuals with alcohol-related injuries of lesser severity. While the 2013 National Health Survey marks the latest effort to evaluate a representative sample of Brazilians regarding health status and associated behaviors (i.e., alcohol use), alcohol-related injuries were not included as a health outcome (8). Individual-level data on SES risk factors for non-fatal alcohol-related injury is important to informing public health strategies aimed at injury control and prevention (12, 13). The objective of this study was to describe the prevalence of alcohol-related non-fatal injuries and analyze its association with demographics and individual-level SES in Maringá, Brazil.

## Methods

### Setting

Our study is a secondary data analysis of alcohol-related injuries collected in a larger cross-sectional study of all cause non-fatal injury conducted in Maringá, Brazil. The parent study, conducted in 2015, used a household survey to examine treatment-seeking behaviors following injury (14). Maringá is the third largest city in the southern state of Paraná. Maringá has a growing population of 375,596 inhabitants, most of whom reside in urban areas (98%) (15). In 2010, Maringá's Human Development Index (HDI) was the second highest in the state (HDI = 0.808) (16).

### Sampling and Population

The sample was derived from a larger cross-sectional study, “Determinants of Care-Seeking Behavior in Maringá, Brazil,” utilizing a household survey to examine treatment-seeking behavior following injury in this population (14). Data was collected between May and September 2015. Cluster-randomized sampling was utilized with a sampling frame that consisted of all households within the city limits.

Sample size was calculated using the population of Maringá at the time of the study (375,596) and an estimated 30% lifetime prevalence of injury (17, 18). With a precision of ± 0.02 and a response rate loss of 5%, the resultant sample size was calculated to be 2017 individuals (19). We calculated the number of individuals needed per census tract for each of the 563 populated census tracts which comprise the city. Census tracts were then stratified by population density and households within each census tract or stratum were randomly chosen for participation.

### Data Collection and Instrument

The study used a modified Surgeons OverSeas Assessment of Surgical Need (SOSAS) survey version 3.0, a validated household survey developed to determine surgical need in LMICs (14, 20). The instrument was modified to collect additional information about injuries.

In each household, the head of the household or primary guardian/caregiver provided informed consent for the household survey in the native local language of Brazilian Portuguese. All household members who spoke Portuguese were eligible for participation. The two-part survey was administered with the first section addressing questions regarding citizenship, birthplace, and years lived in the household. The second section involved the random selection of one individual in the household for a more detailed injury history survey. This randomly selected individual was then required to provide consent again for participation in the second half. If the individual was under the age of 18, a parent or guardian also provided consent. If the individual was under the age of 12, the parent or guardian could answer questions on the child's behalf.

Household surveys were conducted in the native language of Brazilian Portuguese by trained research assistants from Faculdade Ingá, Maringá, Brazil. The first portion of the two-part survey involved demographic information for the whole household and was completed by the head of household. The second part involved a purposive sampling method based on proportions of men, women, elderly, children, and adults in the population. A member of the household was identified and then asked specific questions on personal injury history. Data was collected in Brazilian Portuguese on electronic tablets using REDCap (21). The data was translated from Portuguese to English by bilingual research team members prior to analysis.

### Variables

In our study, injury was defined according to the WHO standard, where an injury is the “physical damage that results when a human body is suddenly or briefly subjected to intolerable levels of energy” (p. 5) (22). Our dependent variable, alcohol-related injury, was defined as self-reported alcohol consumption within 2 h prior to reported injury event. For each injury reported within the last 12 months, participants were asked the follow-up question “Did you consume alcohol 2 h before the injury?” Responses were dichotomized into “yes, alcohol-related injury” and “no, non-alcohol-related injury.”

Our independent variables included demographic variables (age, gender, and race) and socioeconomic variables (employment status, educational attainment, and monthly household income). Age was categorized into under 15 years, 15–29, 30–44, and over 44 years. Participants self-identified into one of the following categories based on their skin color: White, *Pardo*, Black, Indigenous, Asian, Other, or Decline to Respond. Employment was dichotomized into employed and unemployed. Educational attainment was categorized into three categories based on level of schooling completed: at least some primary school completed, at least some secondary school completed, and at least some professional school completed. Monthly household income was rounded to the nearest R$100 and stratified into three categories (low: < R$1,800; medium: R$1,900–R$7,500; high: >R$7,500) as defined by the Organization for Economic Co-operation and Development (23).

### Ethics Statement

We obtained ethical approval from Duke University Institutional Review Board and the Centro Universitário Ingá, Comite de Ética em Pesquisa (CEP, Committee on Ethical Research). This study was associated with minimal risk as participant names and identifying information were not collected.

### Data Analysis

Of the 3,065 households and individuals surveyed, a subgroup of participants who reported an injury was created (*n* = 995). Descriptive analysis was used to describe the distribution of demographic and socioeconomic characteristics of our sample. All study variables were categorical and are presented as frequencies and percentages. We conducted univariate analysis using Chi-square and Fisher's Exact tests and then multivariate analysis using logistic regression models. The age category “Under 15 years” was excluded prior to performing multivariate analyses due to the zero variance of alcohol consumption in this age group. Five cases were missing an alcohol-related injury variable and were excluded from analysis. These exclusions yielded a final sample size of 946 for multivariate analyses. Our reference categories were: over 44 years, female, some primary school complete, identifying as White, employed, and low household monthly income. Odds ratios (OR) with 95% confidence intervals (CI) were calculated for each variable. Associations were considered statistically significant at *p* < 0.05. Data was stored in a REDCap database and all analyses were performed in *R*, version 3.3 (22, 24).

## Results

### Sample Characteristics

Of the 3,065 households and individuals surveyed, the injury prevalence was 32.5% with 995 individuals reporting at least one injury in their lifetime. Characteristics of our study sample are presented in Table 1. The majority were employed (76.8%), over the age of 30 years (68.9%), and identified as being White (62.7%). Nearly half of respondents reported middle income (48.4%), followed by low income (39.8%) and high income (11.8%). Of the 995 respondents who reported injuries, 62 (6.26%) reported alcohol consumption within 2 h of an injury. Males comprised 85.5% of the alcohol-related injuries. Of self-reported alcohol-related injuries, 45.2% respondents reported completing at least some professional-level education, while 21% reported completing at least some primary-level education. Our alcohol-related injury population was 43.5% low-income and 45.2% middle-income, which was similar to our total injury population. While the majority of our alcohol-related injury population identified themselves as White (53.2%), 19.4% of our alcohol-related injury population identified as Black.

**Table 1:** Demographic and socioeconomic characteristics of study sample.

	**Injured *N* = 995**	**Non-alcohol-related injury *N* = 928 (93.2%)**	**Alcohol-related injury *N* = 62 (6.2%)^**b**^**	**Chi Square Statistic *p*-value**
Age				0.004
Under 15 years	44 (4.4)	44 (4.7)	0 (0)	
15–29 years	265 (26.6)	237 (25.5)	26 (41.9)	
30–44 years	260 (26.1)	239 (25.8)	19 (30.1)	
Over 44 years	426 (42.8)	408 (44.0)	17 (27.4)	
Gender				<0.001
Female	472 (47.4)	461 (49.7)	9 (14.5)	
Race				0.049
White	624 (62.7)	587 (63.3)	33 (53.2)	
*Pardo*	229 (23.0)	215 (23.2)	13 (21.0)	
Black	93 (9.3)	81 (8.7)	12 (19.4)	
Other^a^	49 (4.9)	45 (4.8)	4 (6.5)	
Household Monthly Income				0.836
Low	396 (39.8)	369 (39.8)	27 (43.5)	
Middle	482 (48.4)	454 (48.9)	28 (45.2)	
High	117 (11.8)	110 (11.9)	7 (11.3)	
Education				0.235
At least some primary schooling complete	279 (28.0)	266 (28.7)	13 (21.0)	
At least some secondary schooling complete	357 (35.9)	335 (36.1)	21 (33.9)	
At least some professional schooling complete	359 (36.1)	327 (35.2)	28 (45.2)	
Employment status				0.225
Unemployed	231 (23.2)	220 (23.7)	10 (16.1)	

a *“Other” includes Asian, indigenous, other, and decline to respond*.

b *5 cases missing N = 5 (0.5%)*.

### Association of Socio-determinants and Alcohol-related Injury

Table 1 shows the univariate associations of socioeconomic characteristics and demographics for alcohol-related injury. The univariate analysis revealed significant associations between reporting an alcohol-related injury and participant age (Chi-square = 13.06, *p*-value = 0.005), gender (Chi-square = 27.42, *p* < 0.001), and race (Fisher's Exact Test, *p* = 0.039). Multivariate regression analysis results are presented in Table 2. Males were significantly more likely to report an alcohol-related injury than females (OR = 5.98 95% CI = 3.02, 13.28). Compared to participants over 44 years, those aged 15–29 were more likely to report an alcohol-related injury (OR = 3.62 95% CI = 1.72, 7.71). Compared to other races, participants who identified as Black were more likely to report an alcohol-related injury (OR = 2.38 95% CI = 1.09, 4.95). Although employment status was not significantly associated with alcohol-related injury in our univariate analysis (Chi-square = 1.47, *p* = 0.225), in our adjusted model, unemployed participants were less likely to report an alcohol-related injury than employed participants (OR = 0.41 95% CI = 0.18, 0.88).

**Table 2:** Results of multivariable analysis examining demographic and socioeconomic variables associated with alcohol-related injury.

**Demographic and socioeconomic characteristic**	**Adjusted OR**	**95% CI**	***p*-value**
Age			
Over 44 years	*REF*		
15–29 years	3.62	1.72, 7.71	<0.001
30–44 years	1.83	0.19, 0.62	0.100
Gender			
Male	5.98	3.02, 13.28	<0.001
Race			
White	*REF*		
*Pardo*	1.01	0.48, 1.99	0.986
Black	2.38	1.09, 4.95	0.024
Other^a^	2.42	0.67, 6.95	0.130
Household monthly income			
Low	*REF*		
Middle	0.74	0.40, 1.34	0.310
High	0.72	0.26, 1.80	0.503
Education			
At least some primary schooling complete	*REF*		
At least some secondary schooling complete	0.74	0.34, 1.65	0.453
At least some professional schooling complete	1.19	0.89, 3.80	0.675
Employment status			
Unemployed	0.41	0.18 0.88	0.028

a *“Other” includes Asian, indigenous, other, and decline to respond*.

## Discussion

To date, many studies have corroborated a high burden of alcohol-related injury in Brazil and the presence of SES disparities among the injured (3, 5, 11, 25–32). To our knowledge, this study is the first to describe socioeconomic and demographic characteristics of non-fatal alcohol-related injuries, specifically in LMICs, and one of the few looking at non-fatal injuries outside of a large metropolitan area of Brazil. Using household survey data from Maringá/PR, we examined the association of individual-level characteristics with alcohol-related injury. Based on our results, socio-determinant characteristics that were associated with reporting having experienced a non-fatal lifetime alcohol-related injury were age, gender, race, and employment status.

In line with previous research, our study showed gender and age appeared to have the greatest effects on likelihood of reporting having experienced an alcohol-related injury. Males and younger ages (15–29 years) were the most common groups. These findings are consistent with national (Brazilian) and international literature that identified gender and age as significant predictors of alcohol-related injuries, with a greater prevalence of alcohol-related injuries occurring among young men (5, 11, 25–31, 33). Similarly, the most recent Pan American Health Organization report on Alcohol and Health in the Americas highlights that men under the age of 30 represent the largest proportion of patients entering emergency departments with injuries (3, 34). Therefore, it is not surprising that within our study population, the odds of reporting an alcohol-related injury were highest among males and those 15–29 years of age. It is important to note, however, that most literature refers to fatal injuries, usually using hospital-based data. The literature on non-fatal injury is scarce, which highlights the relevance of our study and draws attention to the need to improve public health policy targeted at injury and alcohol use prevention.

The odds of alcohol-related injury among those who identified as Black were observed to be high. In Brazil, very few studies examine racial/ethnic disparities in alcohol-related injury and to date, findings have been inconclusive. Evidence suggest that participants identifying as Black seem to have a higher risk of alcohol-related injury fatality (8, 11, 27, 30, 35, 36). However, recent studies also found that racial/ethnic group did not differ in terms of alcohol positivity in fatally injured patients (26, 37). While there is evidence to support the differential role of race/ethnicity on fatal and non-fatal alcohol-related injuries in LAC countries (38), this relationship has not been researched extensively in Brazil. In the United States, although Blacks have been shown to have lower levels of frequency and amount of alcohol consumption (39, 40), this population has also been associated with a higher risk of injury post–heavy episodic drinking and alcohol dependence (39–43). The United States National Institute of Alcohol Abuse and Alcoholism suggests that US Blacks who drink have a higher frequency of binge drinking and risk of getting involved in injuries (44). Some explanations to this results could be attributed to the effects social/ethnic discrimination leading to alcohol misuse and risk behavior (4, 45, 46), lower socioeconomic status and social disadvantages such as access to highly concentrated alcoholic drinks during binge drinking episodes (e.g., spirits), and greater poverty or inequitable health care treatment (40, 45, 47–49). Blacks, in Brazil, have been consistently suffered from lower quality healthcare and higher mortality, indicating a strong need for an active and enforced public health agenda toward this minority group (38, 49–54).

Individual facets of socioeconomic status, such as income, educational level, and employment, are important determinants of alcohol use and associated harm (4, 55). In HICs, unemployment is usually associated with increased risk for alcohol-related injury (4, 56, 57). However, this association is not definitive and conclusive, as we observed the opposite in our study. We found that participants who were employed were more likely to report an alcohol-related injury than those who were unemployed. Our results are similar to findings in other LMICs with road traffic injuries, such as India, Ghana (32, 58), and Estonia (58). The association between SES and alcohol use, or alcohol-related harm, is complex and dynamic according to contextual characteristics, as it was pointed out in a large review (4). One potential explanation to the results we observed in our study is that exposure to risky behavior for injury after alcohol-use might be linked to greater financial status, such as having a car or drinking in proximity with others such as in bars, parties, or restaurants. Some evidence even supports the notion that alcohol-related injuries are cyclical, happening with higher frequency around the weekends or the end of the month when paychecks are delivered (59, 60). Similarly, evidence suggests that heavy episodic drinking is more common in households with a higher income (4). Injury risk has been shown to increase according to an increase in dosage of alcohol use, more likely to happen in higher income populations.

Similarly, lower educational and income levels have been associated with increased alcohol-injury risk in HICs (4, 5). Whether or not an injury was alcohol-related did not differ across educational or income levels in our study. These findings are in line with a recent study comparing the predictability of demographic factors with alcohol-related injury across countries of varying economic development (5). In the aforementioned study, individual socioeconomic characteristics were important predictors in HICs and lower-middle income countries, but no discernable pattern was observed in upper-middle income countries, including Brazil (5).

### Limitations

This study has several limitations. First, our findings are based on self-reported alcohol-related injury data which are subject to recall and response bias. While under most circumstances, alcohol, and injury self-reporting measures tend to be valid and reliable, there is a tendency in alcohol and injury research toward underreporting (61–63). Similarly, underreporting is possible when reporting socially sanctioned or illegal activities, which could be the situation for study participants under age 18. Alcohol use before 18 years is illegal in Brazil, and it is possible that study participants under age 18 were hesitant to report an injury as alcohol-related. Given these risks for underreporting in our study, the actual number of alcohol-related injuries in our study is likely higher than the 62 reported. Secondly, since the parent study was established to detect a power of overall prevalence of injury among the population, we had a limited sample of alcohol-related injury victims. A larger sample could have revealed associations that were masked due to a small sample size. Our limited sample size also prevented us from conducting additional detailed analyses across subgroups or multi-level analyses. Finally, alcohol-related injury risk is influenced by a multitude of individual-level factors and neighborhood-level factors that work in combination with injury related characteristics (type of injury, cause of injury, place of injury) to influence risk. Neighborhood-level factors and injury characteristics were outside the scope of this study and future studies examining these characteristics are important.

## Conclusion

Notwithstanding the limitations, our results provide new local evidence regarding socioeconomic and demographic characteristics of non-fatal alcohol-related injury in Brazil. Specifically, we identified that being young, employed, males or who identify as Black were independently associated with an increased risk for non-fatal alcohol-related injury in Maringá. Our results also underlined the complex nature of the associations between individual level facets of SES and demographic factors (age, gender, race) with alcohol-related injury. Future studies with larger sample sizes and inclusion of cultural-contextual variables (i.e., injury type, drinking behavior, environment/mechanism of injury) are needed. Mitigating the burden of alcohol-related injuries in a country as vast and diverse as Brazil requires a comprehensive, multifaceted approach. Individual level data, such as ours, should be considered in combination with area-level and country-level data when developing evidence-based public-health policies. Given the health disparities by race/ethnicity groups in Brazil and worldwide, examining the distribution of alcohol-attributable groups that may be at higher risk for alcohol-related injury is important for public health interventions and to provide equitable access to preventive care for alcohol use and injury risky behavior.

## Data Availability Statement

The raw data supporting the conclusions of this article will be made available by the authors, without undue reservation, to any qualified researcher.

## Author Contributions

NT and LA contributed to the conception and design of the study under the supervision of JV and CS. NG, AO, and PC performed data collection and wrote sections of the manuscript. DE-G, YT, SW, and LA conducted statistical analysis under the supervision of JV. DE-G, YT, and SW wrote the manuscript and revised as needed under the guidance of JV and CS. JV and CS supervised the overall study and provided feedback throughout all stages.

### Conflict of Interest

The authors declare that the research was conducted in the absence of any commercial or financial relationships that could be construed as a potential conflict of interest.
